# Short-Course Versus Long-Course Colistin for Treatment of Carbapenem-Resistant *A.* *baumannii* in Cancer Patient

**DOI:** 10.3390/antibiotics10050484

**Published:** 2021-04-22

**Authors:** Wasan Katip, Suriyon Uitrakul, Peninnah Oberdorfer

**Affiliations:** 1 Department of Pharmaceutical Care, Faculty of Pharmacy, Chiang Mai University, Chiang Mai 50200, Thailand; 2 Epidemiology Research Group of Infectious Disease (ERGID), Chiang Mai University, Chiang Mai 50200, Thailand; aoberdor@med.cmu.ac.th; 3 Department of Pharmaceutical Care, School of Pharmacy, Walailak University, Thai Buri 80160, Thailand; Suriyon.ui@wu.ac.th; 4 Division of Infectious Diseases, Department of Pediatrics, Faculty of Medicine, Chiang Mai University, Chiang Mai 50200, Thailand

**Keywords:** cancer patients, duration of treatment, colistin, propensity score analysis, multidrug-resistant *Acinetobacter baumannii*

## Abstract

Carbapenem-resistant *Acinetobacter baumannii* (CRAB) is one of the most commonly reported nosocomial infections in cancer patients and could be fatal because of suboptimal immune defenses in these patients. We aimed to compare clinical response, microbiological response, nephrotoxicity, and 30-day mortality between cancer patients who received short (<14 days) and long (≥14 days) courses of colistin for treatment of CRAB infection. A retrospective cohort study was conducted in cancer patients with CRAB infection who received short or long courses of colistin between 2015 to 2017 at Chiang Mai University Hospital (CMUH). A total of 128 patients met the inclusion criteria. The results of this study show that patients who received long course of colistin therapy had a higher rate of clinical response; adjusted odds ratio (OR) was 3.16 times in patients receiving long-course colistin therapy (95%CI, 1.37–7.28; *p* value = 0.007). Microbiological response in patients with long course was 4.65 times (adjusted OR) higher than short course therapy (95%CI, 1.72–12.54; *p* value = 0.002). Moreover, there was no significant difference in nephrotoxicity (adjusted OR, 0.91, 95%CI, 0.39–2.11; *p* value = 0.826) between the two durations of therapy. Thirty-day mortality in the long-course therapy group was 0.11 times (adjusted OR) compared to the short-course therapy group (95%CI, 0.03–0.38; *p* value = 0.001). Propensity score analyses also demonstrated similar results. In conclusion, cancer patients who received a long course of colistin therapy presented greater clinical and microbiological responses and lower 30-day mortality but similar nephrotoxicity as compared with those who a received short course. Therefore, a long course of colistin therapy should be considered for management of CRAB infection in cancer patients.

## 1. Introduction

Patients with cancer are at-risk of infections caused by antibiotic resistant Gram-negative bacteria. *Acinetobacter baumannii* is one of the most commonly reported nosocomial infections in cancer patients [1]. *A. baumannii* has been identified in patients with solid tumors, hematological malignancies, neutropenia, and those in the Intensive Care Unit (ICU) [2,3,4]. Infections caused by *A. baumannii* can be fatal in patients with suboptimal immune defenses, especially cancer patients [1,2]. Moreover, the prevalence and mortality rate of carbapenem-resistant *A*. *baumannii* (CRAB) has increased. The mortality rate of patients with cancer and multidrug-resistant (MDR) *A*. *baumannii* infection has reached 55% [5].

Colistin treatment for *Acinetobacter baumannii* infection is one of the most debated regimens [6]. Therefore, several alternative treatments are suggested, such as tigecycline, amikacin, and sulbactam. Tigecycline is one of the active antibiotics against CRAB and appears to be a potential alternative therapeutic option for the treatment of CRAB [6,7]. However, tigecycline provides low concentration in plasma, and this limits its use in blood stream infections [6]. Furthermore, tigecycline has been shown to be inferior to the comparator drugs and has shown higher mortality rate in VAP patients [7]. For amikacin and sulbactam, although they have shown anti-CRAB efficacy, their nephrotoxicity and high resistance rate among CRAB limits their use [6]. Therefore, as compared with the other drugs, colistin is still safe and effective for the treatment of CRAB infection [6].

Colistin is one of the most widely prescribed medicines for the treatment of carbapenem-resistant *A. baumannii* (CRAB). It has long been acknowledged that higher consumption of colistin resulted in higher risk of MDR bacteria, as well as higher treatment cost [6]. Theoretically, short-course treatment decreases ecological pressure and eliminates adverse effects without affecting the outcome [8]. Nonetheless, a subgroup of ventilator-associated pneumonia (VAP) patients who were infected with non-maturing Gram-negative microorganisms had higher recurrence of pulmonary infection with the short-course (8 days) treatment regimen than with the long-course regimen [9]. 

The duration of colistin treatment CRAB is typically ≥7 to 14 days [10,11,12]. However, CRAB is known as a significant and difficult-to-treat pathogen with complex resistance. This characteristic is a real challenge to all clinicians and leads to the use of colistin for longer than 2 weeks for established infections in many patients [12].

There was a randomized, open-label, clinical trial that studied 210 patients with life-threatening infections due to extensively drug-resistant (XDR) *A. baumannii*. The recruited patients were randomly assigned to either colistin alone or colistin plus rifampicin groups. The primary end point of the study was overall 30-day mortality. Treatment had to be administered for at least 10 days and up to a maximum of 21 days [12].

However, the optimal treatment duration for a specific group of CRAB-infected cancer patients still remains to be determined. Therefore, the primary objective of this study was to compare clinical outcome, microbiological response, and nephrotoxicity between cancer patients receiving a short course (<14 days) and long course (≥14 days) of colistin for treatment of CRAB. The secondary objective of this study was to compare 30-day mortality rates between patients who received short and long courses of antimicrobial therapy for CRAB pneumonia.

## 2. Results

**Figure 1:**
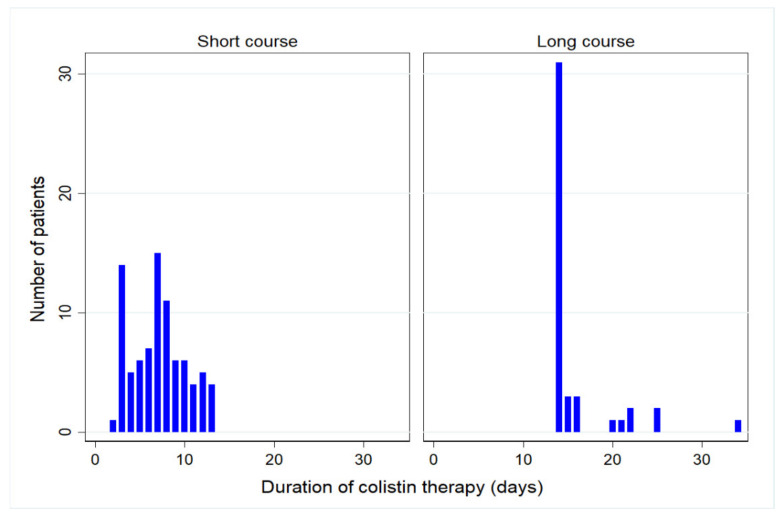
Number of patients who received short and long courses of colistin therapy.

One hundred and twenty-eight cancer patients with CRAB infection were recruited in the study; there were 84 patients in the short course and 44 patients in the long course of colistin therapy. For median duration of therapy, the short-course group had 7 days duration (interquartile range (IQR), 5–9 days) while the long-course group had 14 days duration (IQR, 14–15 days). Overall, the median age was 62 years, and 78 patients (61%) were female. The majority of infectious disease was pneumonia (72%). Patients in the short and long courses of colistin therapy were comparable in most baseline demographics and clinical characteristics, although the numbers of patient in both group were different (Table 1). The number of patients who received short and long courses of colistin therapy is shown in Figure 1.

When assessing the outcomes and toxicity of colistin therapy, Fisher’s exact test showed that the clinical and microbiological response of CRAB infection was higher in the long-course therapy group than in the short-course. There was no significant difference in nephrotoxicity between both patient groups (54 cases (64.29%) in short-course group and 27 cases (61.36%) in long-course group, *p* value = 0.847). Moreover, the 30-day mortality of CRAB infection was higher in the short course than in the long course of colistin therapy group (32 (38.10%) and 5 (11.36%), respectively, *p* value = 0.002), as shown in Table 2.

### Univariate and Multiple Logistic Regression Analyses of the Outcomes

The results of univariate analysis indicate significant differences in clinical response, microbiologic response, and 30-day mortality rates between the short- and long-course groups. Table 3 shows similar nephrotoxicity rates in both groups. However, a significantly higher clinical response rate (odds ratio (OR), 3.16; 95% confidence interval (CI), 1.37–7.28; *p* value = 0.007), higher microbiological response rate (OR, 4.65; 95% CI, 1.72 to 12.54; *p* value = 0.002), and lower 30-day mortality rate (OR, 0.11; 95% CI, 0.03 to 0.38; *p* value = 0.001) were observed in patients who received long-course therapy as compared with short-course therapy based on multiple logistic regression analysis. However, the nephrotoxicity rates were not different (OR, 0.91; 95% CI, 0.39 to 2.11; *p* value = 0.826) (Table 3). Other predictors of clinical response were septic shock and Charlson score ≥ 4, and the predictor of microbiological response was septic shock. Age of 60 years or more could predict higher nephrotoxicity. Other independent risk factors for 30-day mortality were septic shock, Charlson score ≥ 4, and baseline Scr ≥ 1 mg/dl (Table 3).

The results of propensity score analysis using inverse probability weighting with variables associated with long-course therapy showed a significant difference in clinical response, microbiological response, and 30-day mortality rate (*p* value = 0.001, *p* value = 0.001 and *p* value = 0.001, respectively, Table 3). Factors that were used in the analysis were age, sex, vancomycin, amphotericin B, septic shock, Charlson score, comorbidities, stay in ICU during infection, mechanical ventilation during infection, baseline serum creatinine, source of CRAB infection, and type of malignancy.

## 3. Discussion

The results from this study point out that greater clinical and microbiological responses were achieved in cancer patients who received a long-course colistin therapy compared with a short-course. Additionally, no significant difference in nephrotoxicity rate between the two groups was detected. Furthermore, cancer patients who received long-course colistin therapy had lower 30-day mortality rate than the short-course. These findings were supported by logistic regression analysis and propensity score analysis using inverse probability weighting for both primary outcome (i.e., clinical response, microbiologic response, and nephrotoxicity) and secondary outcome (30-day mortality).

Guidelines of antimicrobial therapy are important tools that can help clinicians to make decisions about the duration of treatment. The optimization of antimicrobial duration might also be an important factor for management of CRAB infection in cancer patients. However, prior studies examining the impact of duration of antimicrobial therapy in cancer patients with CRAB infection remain limited. Most randomized controlled trials on duration of antibiotic therapy do not include difficult-to-treat patients or pathogens, such as immunocompromised patients, critically ill patients, specific infection foci, *P*. *aeruginosa* infection, and *A*. *baumannii* infection [13]. Therefore, the optimal duration of antimicrobial therapy for cancer patients with CRAB infection remains uncertain and needs to be investigated. 

In some recent studies, the duration of antibiotic therapy for *Acinetobacter baumannii* infection was not well defined [10,11,12,13,14]. In previous studies, the definition typically used for short-course treatment was less than 10 days, while long-course treatment was typically defined as more than or equal to 10 days [15,16,17]. However, some studies defined treatment of longer than 7 or 8 days as a long-course duration [9,18]. In the clinical practice guidelines by the Infectious Diseases Society of America and the American Thoracic Society (IDSA/ATS), a 7-day course of antimicrobial therapy was strongly recommended for hospital-acquired and ventilator-associated pneumonia rather than a longer duration, even in non-fermentative Gram-negative bacilli infection including *P. aeruginosa* and *A. baumannii* [19]. Remarkably, *A. baumannii* might be one of the most debated pathogens for antibiotic treatment duration; many clinicians usually consider continuation of antibiotic therapy for up to 2 weeks in many patients with established infection [10,11,12,13,14]. However, using antibiotics for 14 days was classified as a long treatment duration in many studies and guidelines [9,10,11,12,14,15,16,17,18,19]. Based on the mentioned information, especially in immunocompromised patients, a 14-day duration was defined as long course therapy; this cut-off was longer than most studies but was set based on the principle of immunocompromised status of patients [9,10,11,12,14,15,16,17,18,19]. Nevertheless, with regard to the patient distribution, where approximately 70% of patients in the long-course group received 14 days of treatment, the effect on the 14-day patients could dominate the effect of other patients. This, therefore, should be considered because the results might be altered if another cut-off were used.

The difference in duration of treatment between 8 and 15 days was analyzed by subgroup in a randomized controlled trial that was conducted in 401 patients with VAP. There was no significant difference in 28-day mortality rate between the two groups with non-fermentative Gram-negative bacilli; the 8-day group had 23.4% and the 15-day group had 30.2% 28-day mortality rates (−6.8% difference; 90% CI: −17.5–4.1). Likewise, no significant difference was observed in clinical response. However, in the 8-day group, the rate of recurrent pulmonary infection was higher than in the other group: 40.6% and 25.4%, respectively (15.2% difference; 90% CI: 3.9–26.6) [9]. Therefore, longer antibiotic therapy might be necessary and should be considered in patients with VAP caused by non-fermentative Gram-negative bacilli (1.7% was *A. baumannii*) [9].

Multiple logistic regression and propensity score analysis showed that patients in this study who received a long course of colistin were more likely than those who received a short course to experience clinical response (61% and 26%, respectively; *p* value = 0.005) and microbiological response (61% and 34%, respectively; *p* value = 0.013). These data further support the notion that longer colistin therapy is highly useful in the treatment of MDR *A. baumannii* in cancer patients.

The results of this study were consistent with the retrospective study by Nelson et al. [20] that included 117 patients with a short course and 294 patients with a long course of antimicrobial therapy (median duration of 8.5 and 13.3 days, respectively) for uncomplicated Gram-negative bloodstream infection. The propensity score adjusted risk of treatment failure was higher in patients with a short course of antimicrobial agents compared with a long course (HR 2.60, 95% CI: 1.20–5.53, *p* value = 0.02). Moreover, the compromised immune status was found to be a risk factor for treatment failure (HR 4.30, 95% CI: 1.57–10.80, *p* value = 0.006). However, the abovementioned study was not conducted using a specific treatment for *A. baumannii* infection, so this result might not be applicable in this pathogen [20].

Another study by Hachem et al. [21] evaluated the efficacy of colistin in cancer patients, mostly patients with hematological malignancy treated for multidrug-resistant *Pseudomonas aeruginosa* infection; it was found that clinical cure rate was 61% and median treatment duration of colistin was 20 days (range from 5–58). However, the objective of this study was not to investigate duration of treatment, so the result found for duration in the Hachem et al. [21] study could not indicate a suitable duration of treatment.

Nazer et al. [22] described that microbiological clearance was observed in 51 patients (66.2%) on day 13 ± 9 (mean ± SD) after starting colistin therapy. They found that 35 out of 89 patients with cancer and CRAB infection (39.3%) met the RIFLE criteria for nephrotoxicity, with a mean of 9 ± 7 days from the initiation of colistin therapy. Duration of IV colistin was 15.8 ± 11 days (mean ± SD). However, the mentioned study was not designed to compare the courses of antibiotic therapy, so there was no control group for comparison [22].

Theoretically, in cases where a patient has good clinical response without clinical features of infection, the duration of antibiotic therapy should be shortened from the traditional 14–21 days (long course) to a period as short as 7 days (short course) because a short-course therapeutic approach can reduce ecological pressure and diminish side effects. However, cancer patients with CRAB in this recent study had lower clinical and microbiological responses when using a short-course treatment regimen. There were several reasons for the requirement of a long course (>14 days) of antibiotic therapy in CRAB-infected patients. First, one of the potential causes of delayed eradication of CRAB from the body might be attributed to chemotherapy administration, which diminishes neutrophil function [23]. Since neutrophils have several essential roles in host resistance to respiratory infection from many organisms as well as from *A. baumannii* [24], cancer patients infected with CRAB should considered for longer antibiotic therapy. Secondly, antibacterial therapy aims to reduce the number of bacteria, but in most cases, antibacterial efficacy relies on the immune system to eliminate bacteria completely. However, patients with malignancy can sometimes have neutropenia and lack of the defense mechanisms that are present in patients with intact immune systems. Therefore, a higher pharmacokinetic (PK)–pharmacodynamic (PD) target and longer antibiotic therapy may be required in immunosuppressed patients such as cancer patients. This hypothesis is supported by the neutropenic mouse thigh model, where it was found that neutropenia increased the required magnitude of PK/PD index by 50% to 100% (i.e., 1.5–2.0-fold) [25,26]. 

The poor outcomes reported in this study might be caused by underlying malignant diseases in patient population, but there were also other potential contributing factors. After adjusting the potential confounders, other predictors of clinical response were detected, including septic shock (OR, 0.3; 95% CI, 0.13 to 0.68; *p* value = 0.004) and Charlson score ≥ 4 (OR, 0.34; 95% CI, 0.16 to 0.74; *p* value = 0.006), and the predictor of microbiological response was septic shock (OR, 0.37; 95% CI, 0.15 to 0.88; *p* value = 0.024). The other independent risk factors for 30-day mortality were septic shock (OR, 6.20; 95% CI, 1.67 to 23.10; *p* value = 0.007), Charlson score ≥ 4 (OR, 7.12; 95% CI, 2.40 to 21.10; *p* value = 0.001), and baseline Scr ≥ 1 mg/dl (OR, 2.85; 95% CI, 1.02 to 7.97; *p* value = 0.046).

In the present study, 54 (64.29%) and 27 (61.36%) patients developed nephrotoxicity during short course and long course therapy (*p* value = 0.847), respectively. The incidence of nephrotoxicity observed in this study was within the range that has been reported in previous studies, ranging from 20% to 69% [27,28,29]. The other factor that was found correlated with nephrotoxicity was age equal or greater than 60 years (OR, 2.31; 95%CI, 1.01–5.33).

Regarding antimicrobial stewardship, reducing the length of antibiotic course might be effective in reducing antibiotic resistance through the abovementioned mechanism. However, this study observed higher rates of clinical and microbiological response, similar nephrotoxicity, and a lower rate of 30-day mortality in the long-course therapy than in short-course therapy. Therefore, in the specific group of cancer patients with CRAB infection, consideration of longer treatment was necessary.

This study differed from other previously reported studies since all patients here had underlying cancer. Eighty-six percent of the patients had solid tumor, and the rest had hematologic malignancy. Additionally, all patients in this study had CRAB infection without any other infections.

There were some limitations in this study. Firstly, the methodology of this study was retrospective, which could possibly have allowed unknown variables to affect the results. However, sensitivity analysis was used to adjust for the suspected confounding factors, and the obtained results still led to the same conclusion. Secondly, in a previous prospective study, the incidence of acute kidney injury (AKI) during therapy was strongly correlated with baseline renal function and plasma colistin concentration. Patients with a higher creatinine clearance (CL_CR_) had higher rates of AKI than those with a lower CL_CR_ [30]. As this study did not measure plasma colistin concentration, there was a lack of pharmacokinetic information, which was considered one of limitations of this study. However, therapeutic drug monitoring, especially for colistin, is not routinely performed in clinical practice in Thailand. RIFLE criteria were therefore used to assess nephrotoxicity outcome. The baseline renal functions were not significantly different between short- and long-course treatment groups. Moreover, logistic regression analysis and propensity score analysis using inverse probability weighting with variables associated with long-course therapy were performed to adjust any variables that were different between short- and long-course treatment groups. Thirdly, this study did not describe the type of chemotherapy that patients might have been administered, so the results of this study should be carefully interpreted based on cancer patient status. Fourthly, this study did not explore patient blood counts, which might affect the outcomes.

## 4. Materials and Methods

This retrospective cohort study was conducted at Chiang Mai University Hospital (CMUH), Chiang Mai, Thailand, from January 2015 to August 2017. The methodology was approved by the ethics committee on human research of the Faculty of Medicine, Chiang Mai University, with a waiver of informed consent for retrospective data collection under the condition of anonymously stored data collected. Medical chart records and microbiology laboratory data of cancer patients with CRAB infection were reviewed. The criteria used to identify and classify infection were outlined by the Center for Disease Control and Prevention (CDC) [31]. The inclusion criteria were age equal or greater than 18 years, colistin treatment for more than 2 days for documented CRAB infection, and receipt of only one course of colistin treatment. The exclusion criteria were the presence of other types of Gram-negative infection, and treatment with hemodialysis or renal replacement therapy. The primary exposure was duration of antibiotic treatment, divided into short-course and long-course therapy. The first day of colistin therapy was defined as the day that CRAB cultures were obtained. Short-course antimicrobial therapy was defined as a total duration of colistin <14 days after the first day. Long-course therapy was defined as a total duration of colistin ≥14 days after the first day. The duration was the first day of intravenous colistin use until fully discontinuation.

The following information was collected: age, sex, type and site of malignancy, comorbidities, dates of admission and discharge, medical history, ICU admission, the need for mechanical ventilation, Charlson score, previous nosocomial infection, clinical course, length of hospital stay, and hospital discharge diagnosis. Data on positive bacterial cultures, antibiotic susceptibilities, colistin minimum inhibitory concentration (MIC), source of CRAB infection, subsequent culture results, and the duration of colistin therapy were also collected. Side effects related to colistin, baseline serum creatinine, baseline GFR, total colistin dose, and the development of septic shock during the evolution of CRAB infection were recorded, as well as the concomitant nephrotoxic medications that could potentially cause nephrotoxicity, hospital discharge diagnosis, outcome, nephrotoxicity (base on RIFLE criteria), and 30-day mortality.

CRAB was defined as *A. baumannii* that was resistant to carbapenems but sensitive to colistin. Dosage of antibiotic regimens was based on the respective hospital guidelines: LD colistin 300 mg of colistin base activity (CBA) once at the start of treatment course and then 150 mg of CBA every 12 h. Colistin administration was adjusted according to renal function. Dose and interval were adjusted according to Cockcroft and Gault creatinine clearance estimates if patients had moderate-to-severe renal impairment (creatinine clearance rate, <50 mL/min). For example, a loading dose of 300 mg followed by maintenance dose of 150 mg CBA every 24 h was administered for creatinine clearance rate of 20–50 mL/min, or 150 mg CBA every 48 h was administered for creatinine clearance rate of <20 mL/min.

### 4.1. Outcome Assessment

Three primary outcomes in this study were clinical response, microbiological response, and nephrotoxicity after treatment. Clinical response of treatment was assessed by resolution or partial resolution of the present symptoms and signs of CARB infection at the end of colistin treatment. Patients who failed to achieve all criteria for clinical response were defined as clinical failures. Microbiological response was defined as obtaining two consecutive negative CARB cultures from the site of infection after the initial positive culture, whereas microbiological failure was defined as persistence of CARB in the subsequent specimen cultures. Renal toxicity was defined as detection of any stage of acute kidney injury outlined in the RIFLE classification [32]. The secondary outcome of the study was 30-day mortality, which was defined as death within 30 days after initial colistin treatment for CARB infection.

### 4.2. Antimicrobial Susceptibility Testing

*A. baumannii* was discovered using traditional cultures and biochemical methods at CMUH’s Clinical Microbiology Division. The Clinical and Laboratory Standards Institute (CLSI) protocol [33] was used to assess antimicrobial susceptibility. Antibiotic susceptibility to *A. baumannii* was determined using the VITEK 2 method, and colistin susceptibility was determined using broth microdilution, with resistance identified as a colistin MIC breakpoint >2 mg/L. The Vitek 2 system (bioMerieux, Marcy I ‘Etoile, France) is a fully automated system that uses a fluorogenic approach to identify organisms and a turbidimetric process to assess susceptibility. Antimicrobial susceptibility testing with VITEK 2 demonstrated high compliance with standard methods for evaluating antimicrobial MICs, with a time benefit of hours to days and increased reproducibility [34,35].

### 4.3. Statistical Analysis

Stata 14 software was used to analyze all of the results (Stata-Corp, College Station, TX, USA). The duration of colistin therapy was mainly compared between the two treatment groups.

General characteristics and basic information of patients were analyzed with descriptive statistics, including percentage, frequency, average, and standard deviation. Using Fisher’s exact test, the average comparison case of sample basic data and the average of other statistical methods was the independent *t* test when data were distributed normally, and the Mann–Whitney U test when data were not normally distributed. The significance level was set as 0.05. Fisher’s exact test was used to compare differences in rates of clinical response, microbiologic response, nephrotoxicity, and 30-day mortality between short-course and long-course colistin therapy.

Furthermore, factors that might affect the four outcomes, i.e., clinical response, microbiologic response, nephrotoxicity, and 30-day mortality, were adjusted using logistic regression. The evaluated factors included age, comorbidities, stay in an intensive care unit (ICU) during infection, course of colistin therapy, septic shock, Charlson score, baseline serum creatinine, type of malignancy, mechanical ventilation during infection, type of nephrotoxic medication, and source of CRAB infection. Firstly, univariate analysis was performed to evaluate the predictive effect of each factor. Next, any factors with a *p* value of < 0.25 from univariate test were included in a full multiple logistic model. Lastly, factors were removed from the model one at a time until all factors remaining in the model had 5% significance level, except that course of colistin therapy remained in the model, regardless of its *p* value.

For sensitivity analysis, a propensity score for courses of colistin therapy and estimated ORs with inverse probability weighting methods was developed using variables likely to influence the outcomes of both primary outcome (i.e., clinical response, microbiologic response and nephrotoxicity) and secondary outcome (30-day mortality).

## 5. Conclusions

The results of this study suggest that a long course of colistin was preferred in the treatment of CRAB infection in a cancer population, based on higher rates of clinical and microbiological response. Furthermore, cancer patients who received a long course of colistin had a lower 30-day mortality rate but the same nephrotoxicity rate. A long course of colistin therapy, therefore, should be considered for the management of CRAB infection in cancer patients.

## Figures and Tables

**Table 1:** Demographic and clinical characteristics of patients in short and long courses of colistin therapy.

Characteristic	Short Course (*n* = 84)	Long Course (*n* = 44)	*p* Value
Sex (no. (%) of patients)			
Male	31 (36.90)	19 (43.18)	0.568
Female	53 (63.10)	25 (56.82)	
Age (years), mean ± SD	61.39 ± 13.44	63.41 ± 14.22	0.431
Type of malignancy, *n* (%)			
Solid tumor	73 (86.91)	38 (86.36)	1.000
- Lung cancer	17 (20.24)	10 (22.73)	
- Brain cancer	9 (10.71)	1 (2.27)	
- Liver, bile duct, and GI cancer	10 (11.90)	3 (6.82)	
- Urogenital cancer	7 (8.33)	6 (13.64)	
- Colon cancer	9 (10.71)	8 (18.18)	
- Bone cancer	6 (7.14)	1 (2.27)	
- Head and neck cancer	6 (7.14)	4 (3.70)	
- Gynecologic cancer	9 (10.71)	5 (11.36)	
Hematologic malignancies	11 (13.09)	6 (13.64)	1.000
- Lymphoma	6 (7.14)	4 (9.09)	
- Leukemia	5 (5.95)	2 (4.55)	
Comorbidities *, *n* (%)	44 (52.38)	23 (52.27)	1.000
- Hypertension	20 (23.81)	13 (29.55)	0.527
- Cardiovascular disease	17 (20.24)	10 (22.73)	0.821
- Diabetes mellitus	7 (8.33)	4 (9.09)	1.000
- Chronic kidney disease	7 (8.33)	1 (2.27)	0.262
- Chronic liver disease	5 (5.95)	0 (0.00)	0.164
- Chronic obstructive pulmonary disease	8 (9.52)	9 (20.45)	0.103
ICU status, *n* (%)	50 (59.52)	28 (63.64)	0.706
Septic shock, *n* (%)	51 (60.71)	27 (61.36)	1.000
Mechanical ventilation, *n* (%)	58 (69.05)	39 (88.64)	0.017
Charlson score, mean ± SD	4.14 ± 2.39)	4.32 ± 2.32	0.691
Baseline SCr, mg/dl, median (IQR)	0.7 (0.5–1.2)	0.9 (0.5–1.6)	0.281
Baseline GFR, ml/min, median (IQR)	94.96 (29.65–114.68)	67.6 (29.83–110.21)	0.394
Baseline GFR < 50, mL/min, *n* (%)	27 (32.14)	17 (38.64)	0.557
Baseline GFR < 20, mL/min, *n* (%)	16 (19.05)	7 (15.91)	0.810
Total CMS dose, g, median (IQR)	1.68 (1.04–2.40)	3.22 (2.10–4.50)	0.001
Meropenem, *n* (%)	25 (29.76)	13 (29.55)	1.000
Concomitant nephrotoxic medications **, *n* (%)			
Aminoglycosides	3 (3.57)	3 (6.82)	0.413
Diuretics	59 (70.24)	37 (84.09)	0.131
Amphotericin B	4 (4.76)	7 (15.91)	0.046
Vasopressor	49 (58.33)	29 (65.91)	0.450
Vancomycin	38 (45.24)	30 (68.18)	0.016
Duration of IV colistin (day), mean ± SD	7.13 ± 2.99	15.82 ± 4.10	0.001
Length of hospital stay (day), median (IQR)	31.5 (20–52)	43.5 (35–57.5)	0.001
Source of CRAB infection, *n* (%)			0.065
Pneumonia	56 (66.67)	36 (81.82)	
Bacteremia	3 (3.57)	3 (6.82)	
UTI	17 (20.24)	2 (4.55)	
Other #	8 (9.52)	3 (6.82)	
Colistin MICs, median (min–max)	0.25 (0.094–1.5)	0.25 (0.064–1.5)	0.853

IV, intravenous; SCr, serum creatinine; GFR, glomerular filtration rate; SD, standard deviation; GI, gastrointestinal tract; UTI, urinary tract infection; IQR, interquartile range. * Patients with > 1 disease; ** Patients prescribed > 1 drug; # Other included intercostal drainage, surgical site infection.

**Table 2:** Overall outcomes and toxicity in short and long courses of colistin therapy.

Outcome	Short Course (*n* = 84)	Long Courses (*n* = 44)	*p* Value
Clinical response	**39** (**46.43**)	**31** (**70.45**)	**0** **.015**
Microbiological response	**49** (**58.33**)	**38** (**86.36**)	**0** **.001**
Nephrotoxicity (RIFLE criteria)	54 (64.29)	27 (61.36)	0.847
- Risk	17 (20.23)	10 (22.72)	
- Injury	17 (20.23)	4 (9.09)	
- Failure	20 (23.83)	13 (29.55)	
- Loss	-	-	
- ESRD	-	-	
30-day mortality	** 32 (38.10)**	** 5 (11.36)**	**0.002**

**Table 3:** The outcomes of cancer patients receiving short-course and long-course colistin for CRAB infection (*n* = 128).

Outcome and Variable	Univariate Analysis			Logistic Regression Analysis			Propensity Score Analysis (IPW)		
	Crude OR	95%CI	*p* Value	Adjusted OR	95%CI for Adjusted OR	*p* Value	OR	95%CI	*p* Value
**Clinical response**									
Long course colistin therapy	**2.75**	**1.26–5.98**	**0.011**	**3.16**	**1.37–7.28**	**0.007**	**1.30**	**1.11–1.52**	**0.001**
Septic shock	**0.35**	**0.16–0.74**	**0.006**	**0.30**	**0.13–0.68**	**0.004**			
Charlson score ≥ 4	**0.39**	**0.19–0.79**	**0.010**	**0.34**	**0.16–0.74**	**0.006**			
**Microbiological response**									
Long course colistin therapy	**4.52**	**1.73–11.86**	**0.002**	**4.65**	**1.72–12.54**	0.002	**1.32**	**1.14–1.52**	**0.001**
Septic shock	**0.38**	**0.17–0.87**	**0.022**	**0.37**	**0.15–0.88**	**0.024**			
**Nephrotoxicity**									
Long course colistin therapy	0.88	0.42–1.87	0.745	0.91	0.39–2.11	0.826	1.02	0.85–1.22	0.861
Age ≥ 60 years	1.72	0.83–3.57	0.146	**2.31**	**1.01–5.33**	**0.049**			
**30 days mortality**									
Long course colistin therapy	**0.21**	**0.07–0.58**	**0.003**	**0.11**	**0.03–0.38**	**0.001**	**0.73**	**0.65–0.83**	**0.001**
Septic shock	**6.26**	**2.29–17.50**	**0.001**	**6.20**	**1.67–23.10**	**0.007**			
Charlson score ≥ 4	**4.53**	**1.95–10.49**	**0.001**	**7.12**	**2.40–21.10**	**0.001**			
Baseline Scr ≥ 1 mg/dl	**2.39**	**1.10–5.22**	**0.028**	**2.85**	**1.02–7.97**	**0.046**			

CI, confidence interval; ICU, intensive care unit; OR, odds ratio; IPW, inverse probability weighting.

## Data Availability

The datasets used and analyzed during the current study are available from the corresponding author on reasonable request.
